# Registry for Acute Coronary Events in Nigeria (RACE‐Nigeria): Clinical Characterization, Management, and Outcome

**DOI:** 10.1161/JAHA.120.020244

**Published:** 2021-12-22

**Authors:** Simeon Isezuo, Mahmoud Umar Sani, Abdullahi Talle, Adeyemi Johnson, Abiodun‐Moshood Adeoye, Mehmet S. Ulgen, Amam Mbakwem, Okechukwu Ogah, Emmanuel Edafe, Philip Kolo, Murtala Nagabea, Rasaaq Adebayo, Eze Nwafor, Folasade Daniel, Muiyawa Zagga, Hayatu Umar, Isa Oboirien, Balarabe A. Sulaiman, Umar Abdullahi, Muhammad Sani Mijinyawa, Farouk Buba, Akinyemi Aje, Henry Okolie, Muhammad Nazir Shehu, Umar Adamu, Akinsanya Olusegun‐Joseph, Ranti Familoni, Nwuriku Chibuzor, Taiwo Olabisi Olunuga, Emmanuel Ejim, Awodu Rasheed Olaide, Dike Ojji, Bushra Sanni, Jane N. Ajuluchukwu, Michael O. Balogun, Ayodele B. Omotoso, Mullasari Ajit, Ayodele O. Falase, RACE‐Nigeria Registry Investigators

**Affiliations:** ^1^ Department of Medicine Usmanu Danfodiyo University & Teaching Hospital Sokoto Nigeria; ^2^ Department of Medicine Bayero University Kano & Aminu Kano Teaching Hospital Kano Nigeria; ^3^ Department of Medicine University of Maiduguri Teaching Hospital Maiduguri Nigeria; ^4^ First Cardiology Consultant Hospital Lagos Nigeria; ^5^ Department of Medicine University College Hospital Ibadan Nigeria; ^6^ Cardiology Unit Nizamiye Hospital Abuja Nigeria; ^7^ Department of Medicine Lagos University Teaching Hospital Lagos Nigeria; ^8^ Department of Medicine Bayelsa Specialist Hospital Yenagoa Nigeria; ^9^ Department of Medicine University of Ilorin Teaching Hospital Ilorin Nigeria; ^10^ Department of Medicine University of Abuja Teaching Hospital Abuja Nigeria; ^11^ Department of Medicine Obafemi Awolowo University Teaching Hospital Complex Ile‐Ife Nigeria; ^12^ Department of Medicine University of Port Harcourt Teaching Hospital Port Harcourt Nigeria; ^13^ Department of Medicine Lagos State University Teaching Hospital Lagos Nigeria; ^14^ Department of Medicine Federal Medical Centre Gombe Nigeria; ^15^ Department of Medicine Federal Medical Centre Katsina Nigeria; ^16^ Department of Medicine Federal Medical Centre Bida Nigeria; ^17^ Department of Medicine Olabisi Onobanjo University Teaching Hospital Sagamu Nigeria; ^18^ Department of Medicine Federal Teaching Hospital Abakaliki Nigeria; ^19^ Department of Medicine University of Nigeria Teaching Hospital Enugu Nigeria; ^20^ Institute of Cardiovascular Disease Madras Medical Mission Chennai India

**Keywords:** acute coronary syndrome, incidence, intervention times, reperfusion mortality, Acute Coronary Syndromes, Coronary Artery Disease, Epidemiology, Race and Ethnicity, Risk Factors

## Abstract

**Background:**

Coronary artery disease was hitherto a rarity in Africa. Acute coronary syndrome (ACS) accounts for coronary artery disease–related morbidity and mortality. Reports on ACS in Africa are few.

**Methods and Results:**

We enrolled 1072 indigenous Nigerian people 59.2±12.4 years old (men, 66.8%) with ACS in an observational multicentered national registry (2013–2018). Outcome measures included incidence, intervention times, reperfusion rates, and 1‐year mortality. The incidence of ACS was 59.1 people per 100 000 hospitalized adults per year, and comprised ST‐segment–elevation myocardial infarction (48.7%), non–ST‐segment–elevation myocardial infarction (24.5%), and unstable angina (26.8%). ACS frequency peaked 10 years earlier in men than women. Patients were predominantly from urban settings (87.3%). Median time from onset of symptoms to first medical contact (patients with ST‐segment–elevation myocardial infarction) was 6 hours (interquartile range, 20.1 hours), and only 11.9% presented within a 12‐hour time window. Traditional risk factors of coronary artery disease were observed. The coronary angiography rate was 42.4%. Reperfusion therapies included thrombolysis (17.1%), percutaneous coronary intervention (28.6%), and coronary artery bypass graft (11.2%). Guideline‐based pharmacotherapy was adequate. Major adverse cardiac events were 30.8%, and in‐hospital mortality was 8.1%. Mortality rates at 30 days, 3 months, 6 months, and 1 year were 8.7%, 9.9%, 10.9%, and 13.3%, respectively. Predictors of mortality included resuscitated cardiac arrest (odds ratio [OR], 50.0; 95% CI, 0.010–0.081), nonreperfusion (OR, 34.5; 95% CI, 0.004–0.221), pulmonary edema (OR, 11.1; 95% CI, 0.020–0.363), left ventricular diastolic dysfunction (OR, 4.1; 95% CI, 0.091–0.570), and left ventricular systolic dysfunction (OR, 2.1; 95% CI, 1.302–3.367).

**Conclusions:**

ACS burden is rising in Nigeria, and patients are relatively young and from an urban setting. The system of care is evolving and is characterized by lack of capacity and low patient eligibility for reperfusion. We recommend preventive strategies and health care infrastructure‐appropriate management guidelines.

**Nonstandard Abbreviations and Acronyms**
ACCESS: Acute Coronary Events—A Multinational Survey of Current Management Strategies
RACE: Registry for Acute Coronary Events
THESUS‐HF: The Sub‐Saharan African Survey of Heart Failure

**Clinical Perspective**

**What Is New?**

- The burden of acute coronary syndrome, a disease that was hitherto considered a rarity in the indigenous African population, is rising in Nigeria.
- In this population, patients with acute coronary syndrome are predominantly middle/upper class young urban dwellers and present late with complications.
- The system of care of acute coronary syndrome is evolving and constrained by a lack of prehospital emergency services, prolonged intervention times, and low patient eligibility and infrastructural capacity for reperfusion. Consequently, major adverse cardiac events are frequent and mortality is high.

**What Are the Clinical Implications?**

- The incidence acute of coronary syndrome has the potential of rising with increasing urbanization in Nigeria.
- Management guidelines that are based on best practices in resource‐endowed nations may not be implementable because of delayed presentation and health care infrastructural deficits.
- Current relative low incidence of ACS provides window of opprtunity for preventive measures focusing on promotion of healthy lifestyles, risk factor management, increased awareness, and early diagnosis. In the long term, local context‐based management guideline and improved capacity for reperfusion are required.

Autopsy and clinical studies between the 1940s and 1970s showed that coronary artery disease (CAD) was a rarity among the African population, with prevalence rates ranging from 0.1% to 1%.^1^, ^2^, ^3^, ^4^, ^5^ Subsequent data from various parts of Nigeria showed that the burden of CAD approximately doubled over a period of 3 decades.^6^, ^7^, ^8^, ^9^ Recent reports demonstrated a slowly rising burden of CAD in Nigeria accounting for 0.9% to 1.6% of medical admissions, 2.8% to 3.4% of cardiovascular diseases, and 1.6% to 8.9% of autopsy‐based causes of out‐of‐hospital deaths.^10^, ^11^, ^12^, ^13^, ^14^ The increasing burden of CAD in Nigeria is corroborated by the THESUS‐HF (The Sub‐Saharan African Survey of Heart Failure) data, where CAD contributed to 8% of the causes of heart failure.^15^ A systematic review involving over 90 000 patients from some African nations indicated that the prevalence of acute myocardial infarction ranged between 0.1% and 10.4%.^16^ The rising burden of CAD in low‐ and middle‐income countries like Nigeria results from epidemiological transition from communicable to noncommunicable diseases.^17^ This is characterized by affluence, increased access to health care, and increased longevity resulting in rising prevalence of traditional risk factors for CAD, especially hypertension, diabetes, obesity, and cigarette smoking.^17^

The CAD burden is projected to disproportionately affect Africa, with a doubling of its mortality by 2030.^18^ Acute coronary syndrome (ACS) is the major contributor to CAD mortality. However, reports on ACS in Africa are few. Only a small proportion of the study populations in the South African arm of ACCESS (Acute Coronary Events—A Multinational Survey of Current Management Strategies) were indigenous Black African people.^19^ Reports on ACS in Nigeria are also limited, retrospective, and single‐center based, with no nationally representative and long‐term outcome data. Furthermore, it is not known if the geographical and sociocultural heterogeneity of the Nigerian population influence the frequency and outcome of ACS. The Registry for Acute Coronary Events in Nigeria (RACE‐Nigeria) is aimed at determining the incidence, peculiarities in the characteristics, management, and all‐cause mortality of ACS.

## Methods

### Study Setting

Nigeria is the most populous nation in Africa, with a population of over 200 million representing 2.3% of the world population, and has an average life expectancy of 57.5 years.^20^ There is rapid urbanization, with only 49.8% of the Nigerian population currently being rural dwellers who are mostly engaged in a traditional physically active lifestyle. Health care services are provided at primary, secondary, and tertiary levels and financed mostly by out‐of‐pocket payment. Tertiary health care facilities are located in urban settlements and serve as referral centers for specialized diseases, including ACS.

### Study Design

The registry was prospective, observational, and multicentered. The study population included Nigerian people with ACS. Inclusion criteria included indigenous Nigerian citizens defined as those whose parents are Nigerian and have been living in Nigeria for the last 18 years preceding enrolment and are ≥18 years old. ACS was diagnosed using standard criteria.^21^ ST‐segment–elevation myocardial infarction (STEMI) diagnosis required angina symptoms, ST‐segment–elevation ≥1 mm in at least 2 adjacent limb leads or ≥2 mm in at least 2 contiguous precordial leads or presumed new‐onset left bundle branch block on 12‐lead ECG, and elevated troponin T level. Diagnosis of non–ST‐segment–elevation myocardial infarction required angina symptoms, ST‐segment depression ≥1 mm or T‐wave abnormalities, and elevated troponin T level. Unstable angina was diagnosed in patients with angina symptoms at rest, ST‐segment depression, or T‐wave abnormalities and normal troponin T level. An algorithm was developed and applied to enable classification of ACS and identification of non‐ACS causes of chest pain (Figure S1).

We excluded patients with ST‐segment–elevation following coronary artery bypass graft (CABG) or percutaneous coronary intervention (PCI). Also excluded were Asian, Arabian, White, Caribbean, Nigerian with mixed races, and those with acquired Nigerian citizenship (citizenship by registration or naturalization) populations.

In determining the sample size, we input that the registry is primarily descriptive with the aim of determining the incidence, characteristics, and outcome of ACS. In addition, we took cognizance of the need for internal multiple subgroup comparison that would be subjected to statistical testing. Adequate sample size within the subgroups is required for the utility and information value of these comparisons. Given these considerations and a precision value of 0.5% at 95% CI, as well as combined attrition and missing data rate of 20%, we estimated a minimum sample size of 850.

### Data Collection

Invitation to participate in the RACE‐Nigeria registry was sent to practicing cardiologists in Nigerian tertiary hospitals through the official mailing list of the Nigerian Cardiac Society, announcement during the society's scientific conferences, personal emails and letters. All patients who met the criteria for a diagnosis of ACS in the study centers were consecutively enrolled into the registry between January 2013 and December 2018. Uniformity in approach to patients' recruitment was ensured through use of an ACS diagnostic algorithm (Table S1). A structured interviewer‐administered pretested questionnaire was used to collect data on patients' demography, risk factors, clinical and laboratory characteristics, treatment, and outcome.

We adapted the method previously used in determining socioeconomic status of adults who were parents of pediatric patients in Nigeria^22^ in classifying patients with ACS into upper, middle, and lower socioeconomic classes based on highest educational attainment and occupation. Educational level was categorized as tertiary (university degree or equivalent), secondary school/primary school, and no formal education. Occupations were categorized into 3 groups, taking into consideration the common ones in Nigeria: skilled workers such as professionals, large scale business owners, contractors, politicians, managers, and chief executives; semiskilled workers such as junior public servants, drivers, carpenters, mechanics, sales people, and security guards; and unskilled workers such as subsistence farmers, petty traders, daily paid laborers, cleaners, and messengers. Those without any occupation were considered as unemployed. The categories of occupation and educational level that constituted upper, middle, and lower socioeconomic classes were specified.

Data on laboratory investigations/procedures including troponin, electrolytes urea and creatinine, packed cell volume, and electrocardiography were collected. Electrocardiography and detailed echocardiography were performed routinely in all participants using standard procedures. Chamber dimensions, left ventricular functions, pulmonary artery pressure, status of cardiac valves, and presence of complications including pericardial effusion, intracardiac clot, cardiac rupture, and ventricular aneurysm were determined. Left ventricular systolic dysfunction was classified as severe (<30%), moderate–severe (30%–35%), moderate (36%–40%), mild (41%–49%), and normal (≥50%). Diastolic dysfunction was classified as normal and abnormal (mild, moderate, and severe) in accordance with the recommendation of American Society of Echocardiography and European Society of Echocardiography.

All completed questionnaires and accompanying ECG tracings were sent to the principal investigator. They were scrutinized for ECG diagnostic criteria of ACS. Completed questionnaires were also crosschecked for inconsistencies, duplications, and outliers. Deliberate proactive measures including user‐friendly questionnaire, pilot study, and feedback to investigators were used to minimize missing data. Furthermore, contact addresses and telephone numbers of patients and their next of kin were obtained for tracing defaulters. Follow‐up was at 30 days, 3 months, 6 months, and 1 year after diagnosis of ACS. Outcome measures included incidence, treatment, mortality (in‐hospital, 30 days, 3 months, 6 months, and 1 year), and major adverse cardiac events including cardiogenic shock, reinfarction, stroke, and resuscitated cardiac arrest. The study definitions, end points, and follow‐up were prospectively set up before the commencement of the study.

### Statistical Analysis

Data obtained were analyzed using the Statistical Package for Social Sciences version 21.0 (IBM, Armonk, NY). Continuous variables were checked for skewedness and presented as means±SD for normally distributed data or median with interquartile range for variables that were not normally distributed. An independent *t* test was used to compare means between 2 groups, whereas analysis of variance was used to compare means of 3 or more groups for normally distributed continuous data. Mann‐Whitney tests were used for comparison of skewed continuous variables. Categorical variables are presented as percentages and compared using a χ^2^ test. Incidence rate was calculated with the number of ACS cases as numerator, whereas the adult Nigerian population that accessed the registry study centers (hospitals) during the study period (2013–2018) served as the denominator.

Binary logistic regression analyses were performed, with all‐cause mortality as the dependent variable. To build our models, we first conducted univariate analysis (single predictor variable versus outcome). Variables with skewed distribution were logarithmically transformed in logistic regression analysis to fulfill the necessity of linearity in the logit. Variables that were entered into the stepwise backward selection procedure were selected from literature based on the opinion of the clinical experts on the study team. Age, age category, sex, socioeconomic status, metabolic syndrome (clustered CAD risk factors), heart rate, blood pressures, type of ACS, blood sugar, renal impairment (serum creatinine >2 mg/dL), left ventricular functions, resuscitated cardiac arrest, cardiogenic shock, and intervention (thrombolysis, PCI, and CABG) were inputted into the model. Variables that achieved a significant level in this analysis were used as independent variables (covariates) in stepwise backward binary logistic regression analysis aimed at determining the independent predictors of all‐cause mortality (dependent variable). Predictor variables with the least significant contributions were removed sequentially until none could be further removed without a statistically insignificant loss of fit. Data of logistic regression analysis are presented as odds ratio (OR) with 95% CI. A *P*<0.05 was considered statistically significant.

Models were tested for interaction of independent variables. We used pairs of predictor variables that are likely to work together (interact) in influencing outcome (dependent variable) in creating interaction terms that were investigated by performing backward selection on them. Interactions were considered significant at *P*<0.1.

### Ethical Considerations

Ethical approval was obtained from the institutional review board (Health and Research Ethics Committee) of Usmanu Danfodiyo University Teaching Hospital, Sokoto, Nigeria. Participating health institutions also obtained ethical approval from their respective institutional review boards. All procedures were in accordance with 2013 Geneva Declaration guidelines. Written informed consent to participate in the study was obtained from the patients. Data collected had an institutional identifier, whereas personal identification was anonymized to ensure confidentiality.

### Registry Management

The registry was endorsed by the Nigerian Cardiac Society and managed by a steering committee and advisory council comprising selected experienced cardiologists of professorial status and trainers in the teaching hospitals. The principal investigator appointed institution‐based coordinators who led the data collection in their respective participating hospitals. Personal scientific endeavor was the main motivation for participation. Progress reports on the registry including data collection and analyses were presented during annual scientific conferences of the society between 2013 and 2018. The data that support the findings of this study are available from the corresponding author upon reasonable request.

### Definitions

Type 2 diabetes was diagnosed as fasting blood glucose >126 mg/L (7 mmol/L) or 2‐hour glucose level >200 mg/L (11.1 mmol/L) or history of diabetes. Hypertension was defined as history of hypertension/antihypertensive medication and/or blood pressure persistently ≥140/90 mm Hg. Patients with a high‐density lipoprotein cholesterol level <40 mg/dL (men) or <50 mg/dL (women), and triglyceride level >150 mg/dL, serum total cholesterol >200 mg/dL, or low‐density lipoprotein cholesterol >130 mg/dL were considered to have dyslipidemia. Major adverse cardiac events included reinfarction, stroke, resuscitated cardiac arrest, cardiogenic shock, major bleeding, and death. Myocardial reinfarction was defined as new troponin elevation with either new‐onset chest pain/discomfort or ECG changes consistent with infarction or both. Stroke was defined as focal neurological deficit of vascular origin lasting >24 hours and confirmed by computed tomography scan. Cardiac arrest was defined as recovery following sustained ventricular tachycardia or ventricular fibrillation or asystole.

## Results

### Registry Participants

**Figure 1:**
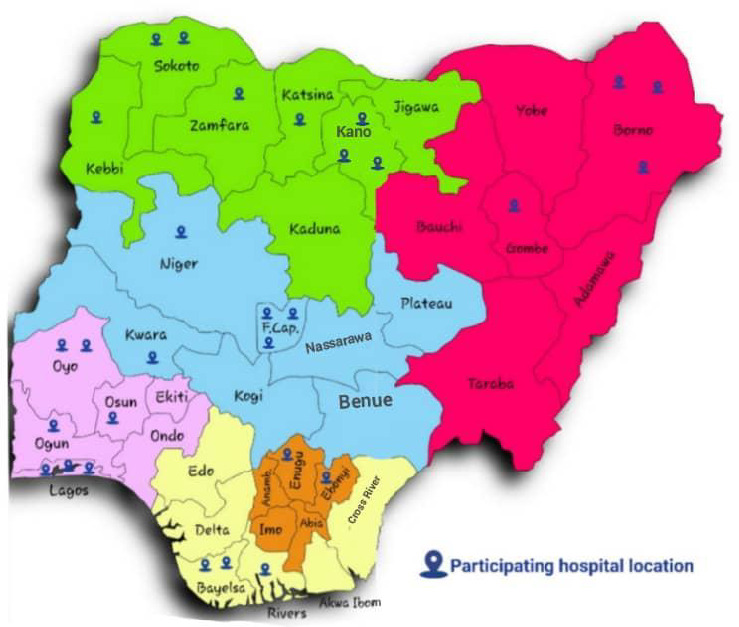
Map of Nigeria showing the distribution of participating hospitals in the 6 geopolitical zones.

**Figure 2:**
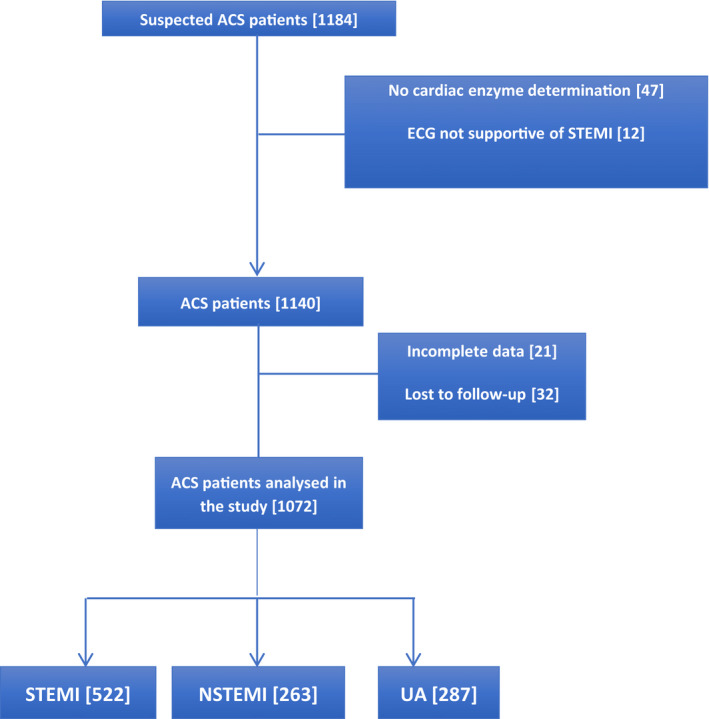
Recruitment of patients with ACS flowchart. ACS indicates acute coronary syndrome; NSTEMI, non–ST‐segment–elevation myocardial infarction; STEMI, ST‐segment–elevation myocardial infarction; and UA, unstable angina.

Twenty‐one (19 public and 2 private) tertiary‐care hospitals constituting 80% of the federal teaching hospitals from all of the 6 geopolitical zones of Nigeria participated in the registry (Figure 1) between 2013 and 2018. A total of 1184 patients were recruited. Of these, 1072 (90.5%) patients from the public (61.7%) and private (38.3%) hospitals were analyzed after excluding patients with the wrong diagnosis of ACS and those with incomplete data or who were lost to follow‐up (Figure 2).

### Incidence

The patients with ACS comprised STEMI (522, 48.7%), non–ST‐segment–elevation myocardial infarction (263, 24.5%), and unstable angina (287, 26.8%), with incidence of 45.98 people per 100 000 hospitalized adults per year. It was higher in the northern than southern part of Nigeria (59.1 versus 22.2 people per 100 000 hospitalized adult patients per year). ACS constituted 1.2% of medical admissions in the public multispecialty hospitals.

### Patients' Characteristics

The sociodemographic and clinical characteristics of the patients are shown in Table 1. They were 59.2±12.4 years old (range, 35–92 years), predominantly men (66.8%), from urban areas (87.3%), and in the upper/middle socioeconomic class (81.7%). Patients in the 50‐ to 69‐year‐old age group were most frequently affected (57.9%). Male patients dominated in all of the age groups except <40 years old and ≥80 years old when both sexes were equally affected. Men peaked about a decade earlier than women in frequency of ACS (50–59 versus 60–69 years old). Transportation of patients to the hospital was mostly with private (769, 71.7%) and commercial vehicles (178, 16.6%), with only 125 (11.7%) being transported by ambulance. The time interval from onset of symptoms to presentation in patients with STEMI was 22.5±46.7 hours (median, 6 hours; interquartile range, 20.1 hours), and only about 3.1% and 11.9% presented within 1 and 12 hours of onset of symptoms, respectively.

**Table 1:** Sociodemographic Characteristics of Patients with Acute Coronary Syndrome

Parameter	Frequency, N=1072
	N (%)
Men	716 (66.8)
Socioeconomic class	
Upper	426 (39.7)
Middle	450 (42.0)
Lower	196 (18.3)
Urban dwelling	936 (87.3)
Transportation by ambulance to the hospital	125 (11.7)
Risk factors*	
Hypertension	844 (78.7)
Dyslipidemia	834 (77.8)
Diabetes	397 (37.0)
Obesity	324 (30.2)
Metabolic syndrome	398 (37.1)
Alcohol	305 (28.5)
Cigarette smoking	262 (24.4)
Family history of MI	197 (18.4)
Clinical presentation*	
Angina	904 (84.3)
Palpitation	422 (39.4)
Diaphoresis	335 (31.3)
Pulmonary edema	238 (22.2)
Nausea/vomiting	174 (16.2)
Epigastric pain/dyspepsia	162 (15.1)
Syncope	122 (11.4)
Vertigo	27 (2.5)
Age, y, mean±SD	59.2±12.4

MI indicates myocardial infarction.

^*^Multiple responses.

**Figure 3:**
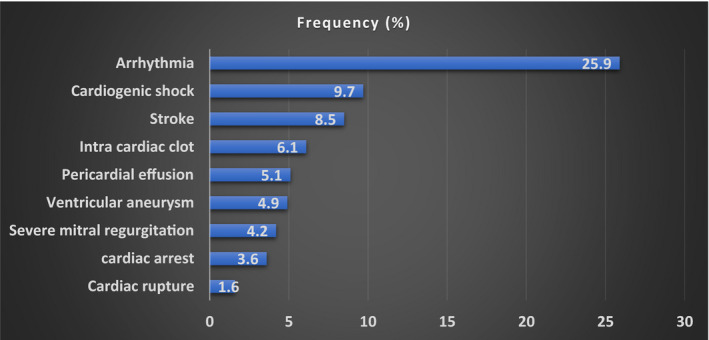
Complications of acute coronary syndrome in Nigerian patients.

The risk factors of ACS are shown in Table 1. Traditional risk factors were observed with clustering as metabolic syndrome demonstrable in 398 (37.1%) patients. About 15.1% (162) of patients presented with atypical symptoms (epigastric pain/dyspepsia). The most frequent complications included arrhythmia (278, 25.9%), pulmonary edema (238, 22.2%), and cardiogenic shock (104, 9.7%) (Figure 3). Frequently observed arrhythmias were atrial fibrillation, sinus bradycardia, ventricular tachycardia, and complete heart block in 97 (9.4%), 64 (6.0%), 57 (5.3%), and 22 (2.2%) patients, respectively. Compared with men, women represented a lower proportion of patients who were in the upper socioeconomic class (33.7% versus 42.9%, *P*<0.0001), smoked cigarettes (6.5% versus 32.0%, *P*<0.0001), and drank alcohol (13.5% versus 34.1%). Female patients also had higher body mass index (30.1±9.2 versus 28.0±5.2, *P*=0.001) and presented more frequently with atypical symptoms than male patients (18.8% versus 13.3%, *P*=0.045).

Compared with patients with non–ST‐segment–elevation myocardial infarction and unstable angina as a group, those with STEMI (Table 2) were younger (58.4±12.2 versus 60.1±12.6 years, *P*=0.04), had a higher proportion of men than women (74.7% versus 59.6%, *P*=0.001), had a higher heart rate (89.8±21.3 versus 86.8±21.0 bpm, *P*=0.03), and had lower left ventricular ejection fraction (45.8±12.9% versus 50.3±29.3%, *P*=0.002). Patients with STEMI also had a higher frequency of cardiogenic shock (11.5% versus 8.9%, *P*=0.01), whereas the non–ST‐segment–elevation myocardial infarction and unstable angina group had a higher frequency of stroke (12.1% versus 7.5%, *P*=0.045) (Table 2). ECG‐determined sites of infarction in patients with STEMI included anterior (56.5%), inferior (28.7%), septal (23.0%), lateral (19.5%), and posterior walls (2.9%), with many patients having multisite involvement.

**Table 2:** Comparison of STEMI and NSTEMI/UA Group

Parameter	STEMI, N=522, N (%)	NSTEMI and UA, N=550, N (%)	*P* value*
Men	388 (74.7)	338 (59.6)	0.001^†^
Hypertension	444 (85.1)	400 (72.7)	0.050
Diabetes	204 (39.1)	193 (35.1)	0.878
Cigarette smoking	147 (28.2)	115 (20.9)	0.129
Alcohol	179 (34.3)	126 (24.6)	0.002^†^
Dyspepsia	85 (16.3)	77 (14.0)	0.888
Multivessel disease	80 (37.2)	74 (44.8)	0.165)
Cardiogenic shock	55 (11.5)	49 (8.9)	0.01^†^
Stroke	35 (7.5)	56 (12.1)	0.045

	Mean±SD	Mean±SD	*P* value^‡^
Age, y	58.4 (12.2)	60.0 (12.6)	0.038^†^
DBP	83.4 (16.6)	80.8 (15.8)	0.011^†^
Duration of HBP	13.2 (9.5)	15.3 (10.8)	0.008^†^
HR	89.8 (21.3)	86.8 (21.0)	0.024^†^
LVEF	45.8 (12.9)	50.3 (29.2)	0.002^†^

DBP indicates diastolic blood pressure; HBP, hypertension; HR, heart rate; LVEF, left ventricular ejection fraction; NSTEMI, non–ST‐segment–elevation myocardial infarction; STEMI, ST‐segment–elevation myocardial infarction; and UA, unstable angina.

^*^ *P* value derived from χ^2^ test.

^†^ *P* value is significant.

^‡^ *P* value derived from independent *t* test.

### Patient Management

The coronary angiography (CAG) rate was 42.4%. Compared with patients who had no CAG, those who had CAG included a higher proportion of patients in the upper socioeconomic class (63.7% versus 23.5%, *P*<0.0001), were >60 years old (54.7% versus 47.8%, *P*=0.03), and had diabetes (42.9% versus 33.9%, *P*=0.004). They also had higher left ventricular ejection fraction (50.3±29.0 versus 45.9±14.0, *P*=0.01) and shorter duration of admission (5.6±3.8 versus 8.6±7.9 days, *P*<0.0001). Sex (*P*=0.248) and type of ACS (*P*=0.245) did not influence CAG rate. Frequently observed culprit vessels included left anterior descending (368, 81.1%), right coronary (215, 47.3%), left circumflex (179, 39.4%), diagonal (83, 18.3%), left main coronary (81, 17.8%), septal (49, 10.8%), and ramus arteries (33, 7.3%). Single‐, double‐, and triple‐vessel involvements were observed in 40.0%, 32.0%, and 28.0% of patients, respectively. The type of ACS did not influence the number of diseased vessel (*P*=0.12).

**Figure 4:**
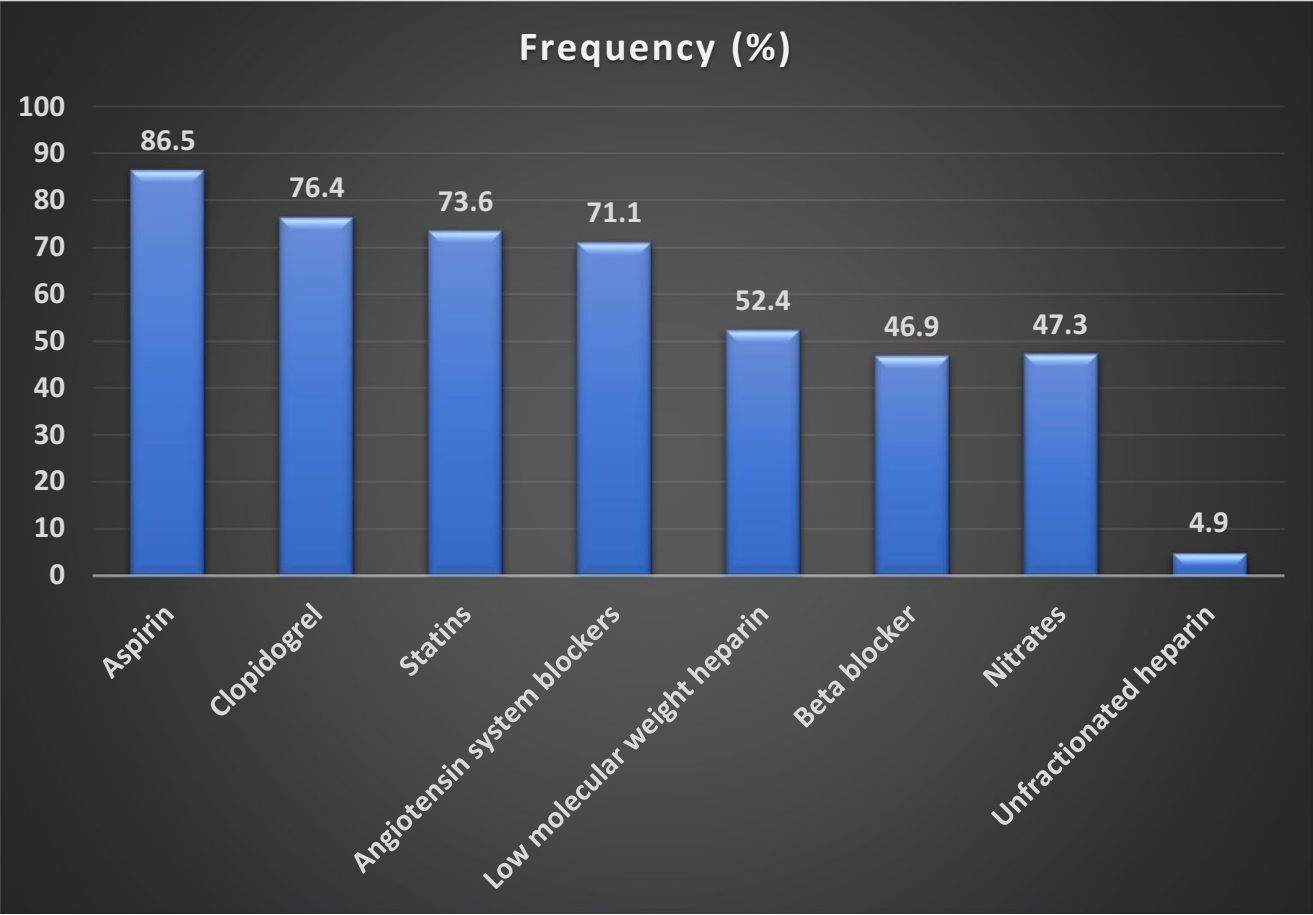
In‐hospital pharmacotherapy of acute coronary syndrome in Nigeria.

A total of 295 (27.5%) patients had preadmission antiplatelet therapy. They were all referred from the first hospital contact and constituted 62.9% of this patient category. Preadmission antiplatelet medications comprised aspirin (163, 55.3%) and clopidogrel (18, 6.1%). In‐hospital antiplatelet treatment included aspirin (927, 86.5%), clopidogrel (819, 76.4%), and dual antiplatelet therapy (887, 82.7%). Other pharmacotherapy including guideline‐based medicines are shown in Figure 4.

Of the 522 patients with STEMI, 89 (17.1%) had thrombolysis using streptokinase (54.0%), tenecteplase (23.8%), and alteplase (22.2%). PCI of all types and CABG were performed in 307 (28.6%) and 120 (11.2%) patients, respectively. Overall reperfusion rate (thrombolysis, PCI, and CABG) was 42.8% and was significantly higher among patients in the upper compared with middle/lower class (65.9% versus 45.6%, *P*<0.0001), with STEMI than non–ST‐segment–elevation ACS (47.7% versus 38.4%, *P*=0.003), with diabetes than without diabetes (47.5% versus 39.9%, *P*=0.020), and patients ≥60 years old compared with those who were younger (46.6% versus 38.9%, *P*=0.014). Sex did not influence reperfusion rate.

### Outcome of ACS

The duration of admission was 7.0±6.4 days, and 19.1% of patients had repeat admissions. Composite major adverse cardiac events were observed in 329 (30.8%) patients, and comprised death in 143 (13.4%), stroke in 91 (8.5%), reinfarction in 57 (5.3%), and resuscitated cardiac arrest in 38 (3.6%) patients. In‐hospital mortality rate was 8.1%. Mortality at 30 days, 3 months, 6 months, and 1 year were 8.7%, 9.9%, 10.9%, and 13.3%, respectively.

Compared with survivors, the deceased patients (Table 3) had significantly higher proportions of patients in the low socioeconomic class (32.9% versus 16.1%, *P*<0.0001) and with STEMI (62.9% versus 46.5%, *P*<0.0001), renal impairment (31.9% versus 13.5%, *P*<0.0001), positive troponin status (85.7% versus 72.3%, *P*<0.0001), diastolic dysfunction (65.5% versus 63.8%, *P*<0.002), pulmonary edema (47.8% versus 21.2%, *P*<0.0001), cardiogenic shock (30.6% versus 7.4%, *P*<0.0001), intracardiac clot (15.3% versus 5.5%, *P*<0.0001), and resuscitated cardiac arrest (21.1% versus 1.2%, *P*<0.0001). Systolic and diastolic blood pressures (*P*<0.0001 each), left ventricular ejection fraction (*P*<0.0001), serum creatinine (*P*<0.0001), and packed cell volume (*P*<0.0001) were significantly lower, whereas heart rate was significantly higher (*P*<0.0001) among the deceased than survivors (Table 3). CAG rate was higher among the survivors than the deceased (49.2% versus 22.9%, *P*<0.0001). The survivors had significantly higher proportion of reperfusion therapy (thrombolysis, PCI, or CABG) than the deceased (94.3% versus 5.7%, *P*<0.0001).

**Table 3:** Comparison of Alive and Deceased Patients with Acute Coronary Syndrome

Parameter	Alive, N=929, N (%)	Deceased, N=143, N (%)	*P* value*
Male	620 (66.7)	96 (67.1)	0.926
Age ≥60 y	475 (51.1)	75 (52.4)	0.995
Low socioeconomic class	149 (16.1)	47 (32.9)	<0.0001^†^
Family history of CAD	185 (21.5)	12 (9.4)	0.001^†^
Metabolic syndrome	354 (47.6)	44 (41.5)	0.241
Atypical symptoms (epigastric pain/dyspepsia)	130 (14.1)	33 (23.2)	0.01^†^
Troponin positive	505 (72.3)	72 (85.7)	0.009^†^
STEMI	432 (46.5)	90 (62.9)	<0.0001^†^
Renal impairment	107 (13.2)	36 (31.9)	<0.0001^†^
Diastolic dysfunction	527 (63.8)	79 (65.5)	0.002^†^
Multivessel disease	209 (38.3)	20 (48.0)	0.194
Pulmonary edema	174 (21.2)	64 (47.8)	<0.0001^†^
Cardiogenic shock	60 (7.4)	44 (30.6)	<0.0001^†^
Intracardiac clot	45 (5.5)	20 (15.3)	<0.0001^†^
Resuscitated cardiac arrest	10 (1.2)	28 (21.1)	<0.0001^†^
Stroke	174 (9.1)	17 (12.9)	0.171
Intervention (thrombolysis/PCI/CABG)	409 (44.0)	107 (18.8)	<0.0001^†^

	Mean (SD)	Mean (SD)	*P* value^‡^
HR	83.8 (19.1)	100.7 (25.2)	<0.0001^†^
SBP	135 (24.2)	120.4 (27.5)	<0.0001^†^
DBP	81.9 (15.3)	76.8 (17.9)	<0.0001^†^
PCV	40 (5.5)	37.6 (7.8)	<0.0001^†^
LVEF	49.9 (26.7)	37.4 (12.6)	<0.0001^†^

CABG indicates coronary artery bypass graft; CAD, coronary artery disease; DBP, diastolic blood pressure; HR, heart rate; LVEF, left ventricular ejection fraction; PCI, percutaneous coronary intervention; PCV, packed cell volume; SBP, systolic blood pressure; and STEMI, ST‐segment–elevation myocardial infarction.

^*^ *P* value derived from χ^2^ test.

^†^ *P* value is significant.

^‡^ *P* value derived from independent *t* test.

Factors that increased the odds of all‐cause mortality on univariate analysis are shown in Table S1. On multivariate logistic regression analysis, mortality increased significantly among patients with diastolic dysfunction compared with those without (OR, 4.1; 95% CI, 0.091–0.570; *P*=0.002), those with pulmonary edema compared with those without (OR, 11.1; 95% CI, 0.020–0.363; *P*=0.001), and those who had resuscitated cardiac arrest compared with those without (OR, 50.0; 95% CI, 0.010–0.081; *P*=0.001). As left ventricular systolic dysfunction progressed in severity, mortality increased by 2.1 times (OR, 2.1; 95% CI, 1.3–3.4; *P*=0.002). Every unit rise in heart rate increased the likelihood of death (OR, 1.037; 95% CI, 1.009–1.065; *P*=0.009). Patients in the lower socioeconomic class were 3.1 times more likely to die than those in the upper socioeconomic class (OR, 3.1; 95% CI, 1.585–6.227; *P*=0.001). Patients without any form of reperfusion therapy (thrombolysis, PCI, and CABG) had 34.5 times likelihood of mortality compared with those who had reperfusion therapy (OR, 34.5; 95% CI, 0.004–0.221; *P*=0.029).

On the effects of independent variables on outcome (all‐cause mortality), statistical interactions were observed between pulmonary edema and left ventricular ejection fraction (Wald, 59.808; *df*, 1; *P*<0.001), cardiogenic shock and left ventricular ejection fraction (Wald, 63.529; *df*, 1; *P*<0.001), ACS type and left ventricular ejection fraction (Wald, 32.161; *df*, 1; *P*<0.001), socioeconomic status and coronary angiography (Wald, 30.658; *df*, 1; *P*<0.001), and systolic blood pressure and pulmonary edema (Wald, 51.83; *df*, 1; *P*<0.001).

### Impact of the Registry

**Figure 5:**
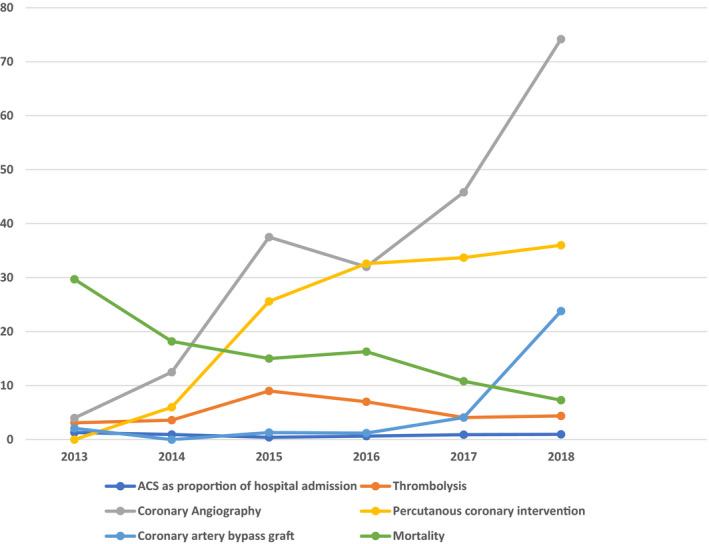
Trends in the burden, management, and outcome of acute coronary syndrome (ACS) in Nigeria.

At the beginning of the registry, only the 2 participating private hospitals had limited capacity for PCI. As the registry progressed, 2 public hospitals developed limited capacity for PCI in collaboration with visiting foreign expertise. Although only 8 hospitals had a facility for biochemical cardiac marker (troponin) at the inception of the registry, all participating centers rapidly acquired this facility during the early period of the registry. More importantly, the rates of CAG, PCI, and CABG increased, whereas the mortality rate fell over the 6‐year period of the registry (Figure 5).

## Discussion

Cardiovascular diseases are the leading causes of death worldwide^23^ and the second leading causes of death in Africa.^24^ CAD accounts largely for cardiovascular deaths^23^ and is projected to disproportionately affect developing nations including Africa.^18^ ACS is the major form of presentation and cause of mortality in CAD. In this pioneer prospective observational, multicenter ACS registry in Nigeria, one of the largest in sub‐Saharan Africa, we characterized indigenous African population with ACS and described their management and outcome. Our ACS population was predominantly in the upper/middle socioeconomic class and from an urban setting. This contrasts the generality of the Nigerian population, in whom over 60% are in the lower socioeconomic class.^20^ Our results demonstrate the rising burden of ACS and evolving systems of care.

The current burden of hospital admissions for ACS suggests an increment by a factor of 20 000 over a period of 60 years with a rise from 0.00005% (1961–1970) in southwestern Nigeria,^5^ through 0.34% (1983–2004) in northeastern Nigeria,^10^ to 0.9% (2000–2005) in northwestern Nigeria.^11^ A recent systematic review of ACS in Africa also showed rising hospital‐based prevalence ranging from 0.1% to 10.4%,^15^ the values being higher in cardiac specialized care‐based studies.^13^ However, our ACS burden is low compared with White^25^ and Asian populations.^26^ This difference may not be unrelated to traditional African lifestyle characterized by high physical activity and intake of high‐fiber diets. Given that our patients are mostly form urban areas, it could be reasoned that urbanization, which increased from 42.6% to 51.2% between 2009 and 2019,^17^ may be the dominant driving factors of ACS in Nigeria. Urbanization has been associated with epidemiologic transition from communicable to noncommunicable diseases, including increased risk factors of cardiovascular diseases, which in turn increased the risk of CAD and ACS.^27^

Interestingly, we observed variation in the incidence of ACS in Nigeria, the value being higher in the northern part of the country. However, the explanatory factors are uncertain. Data from systemic reviews and meta‐analysis, though limited and underpowered, show that the prevalence of diabetes, hypertension, and obesity are higher in the southern than northern parts of Nigeria.^28^, ^29^, ^30^ The prevalence of cigarette smoking, on the other hand, has an opposite trend.^31^ There might be possible contributions of Nigerian ethnic, cultural, and dietary heterogeneity. Cattle rearing, for example, is one of the leading economic sectors among northern Nigerians, and consumption of red meat in smoked (suya), cooked, and fried forms is popular, especially among the urban middle/upper class dwellers in this population.^32^ Consumption of red meat has been associated with ACS, and this is attributable to the rise in saturated fats, arachidonic acid, and heme iron.^33^ Arachidonic acid is a precursor of proinflammatory eicosanoids such as thromboxane A2, which stimulates platelet aggregation, whereas heme iron promotes oxidation of low‐density lipoprotein.^33^ However, the registry did not investigate dietary intake or food frequency.

The clinical characteristics of our ACS population, including traditional risk factors of CAD, are similar to the findings among other African,^34^, ^35^, ^36^, ^37^, ^38^, ^39^, ^40^, ^41^ Asian,^26^ and White^25^ studies. However, our patients are about one and a half decades younger than the White patients, perhaps, because of shorter lifespan and probable earlier onset of risk factors and atherosclerosis among the Nigerian population. We observed an early peak and higher frequency of ACS in men than women. This is similar to previous observations in most races^25^, ^26^, ^35^, ^36^, ^37^, ^38^, ^39^, ^40^, ^41^ and attributable to hormone‐induced protective effects against CAD in premenopausal women.^42^ In addition, we observed a lower frequency of cigarette smoking and alcohol consumption among women. Our female patients also had a higher frequency of atypical symptoms. This may potentially increase the tendency for delayed presentation and underrecognition of ACS in this subpopulation. STEMI was, like most other reports from Africa,^34^, ^36^, ^38^, ^40^, ^41^, ^42^, ^43^, ^44^ the most frequent type of ACS.

Though our ACS population shares some similarities in clinical characteristics and risk factor profiles with White populations, there are important peculiar challenges to myocardial reperfusion. These challenges are common in resource‐constrained nations and linked to ignorance, poverty, lack of a universal health insurance system, inadequate skilled manpower, and deficient health care infrastructure.^34^, ^35^, ^36^, ^37^, ^38^, ^40^, ^41^, ^42^, ^43^, ^44^, ^45^ Consequently, we observed patient‐ and system‐related delays in presentation and low rates of reperfusion, especially thrombolysis and PCI. Sadly, cheap first‐generation nonspecific thrombolytic agents such as streptokinase, which is not a prioritized thrombolytic option in standard guidelines, is hardly affordable in Nigeria. Furthermore, health care financing is mainly by out‐of‐pocket payment, because <5% of the population is covered by the national health insurance system, which does not currently cover thrombolytic agents and CAG, PCI, and CABG procedures.^46^

These constraints, especially delayed presentation and lack of capacity for appropriate reperfusion, are negatively impactful in the management of ACS, particularly STEMI. We also observed a possible gap between ideal guideline‐directed care and practice. A few patients with STEMI presenting beyond the recommended 12‐hour time window were, for example, thrombolysed. This may be attributable to lack of alternative reperfusion options or thrombolysis of patients with hemodynamic instability and/or ongoing ischemia after the recommended window period.

In contrast to reperfusion therapies, the use of guideline‐directed pharmacotherapy compares favorably with reports from other African^19^, ^37^, ^39^ as well as Asian^26^, ^47^ and White^48^, ^49^ studies. However, prehospital emergency medical services are practically nonexistent in Nigeria. Consequently, the proportions of patients who had ambulance services and prereferral antiplatelet therapy are low. Effective adjunct antiplatelet therapy, such as prasugrel, ticagrelor, and glycoprotein P IIa/IIIb antagonists, is hardly used because of the low rate of primary PCI and high cost. Similarly, anticoagulant use is limited to readily available and affordable low‐molecular‐weight and unfractionated heparin.

Given the low patients' eligibility and lack of health care infrastructural capacity for myocardial reperfusion, it is not surprising that our mortality profiles were higher than values from South Africa,^19^ Asia,^26^, ^47^ and Europe.^48^, ^49^ However, they compare favorably with values ranging from 7.8% to 10.5% in some African countries^34^, ^35^, ^39^ and lower than others ranging from 14% to 20%.^37^, ^44^, ^45^ The determinants of ACS outcome are similar to other African,^37^, ^39^, ^45^ Asian,^26^, ^39^, ^47^ and White^48^, ^49^ study data. Left ventricular dysfunction and socioeconomic class are, for example, documented independent predictors of ACS outcome.^50^, ^51^, ^52^ The latter is particularly applicable in the Nigerian setting, which lacks a functional and large‐scale universal health care system. The results of statistical interactions between independent variables that were investigated as possible determinants of ACS outcome would suggest that they were potential effect modifiers of all‐cause mortality.

Our results suggest that the registry had positive impact on the outcome of ACS partly through stimulation of improved health care infrastructure and patient management. There was, for example, improvement, albeit minimal, in noninvasive and invasive diagnostic and therapeutic facilities during the 6‐year period of the registry. This was more pronounced in the private than public sector. The registry might also have encouraged improvement in the basic competency of participating health care providers in the management of ACS. These impacts might explain the observed fall in mortality of ACS over the years as the registry progressed.

The impacts of the registry notwithstanding, RACE‐Nigeria is not without limitations. The registry is hospital‐based, thereby limiting the generalization of our results. ACS incidence and mortality might also have been underestimated because of unreported out‐of‐hospital ACS‐related deaths and exclusion of suspected cases of ACS in health care settings with no facility for troponin assay. Furthermore, there is the possibility of preclusion of patients, especially those from rural areas, who cannot access or afford health care. We also acknowledge that the historical trends of the ACS burden in Nigeria is limited by changing criteria for ACS diagnosis over the years. Finally, patients were lost to follow‐up or excluded because of incomplete data, but this constituted <5% of confirmed patients with ACS.

In spite of these limitations, our findings have implications for Nigeria and sub‐Sahara Africa. First, ACS affects mainly the economically productive age group with potential for strong negative impact on the economy and other critical sectors. Furthermore, increasing urbanization, a common feature of developing nations, may further drive increased ACS burden. Finally, the current relatively low incidence of ACS provides a window of opportunity to institute effective and comprehensive preventive strategies against its rise to an epidemic proportion.

## Conclusions

Our results demonstrate the rising burden of ACS in an indigenous African population that shares similar traditional risk factors of CAD with White populations, although they are relatively younger and present late with complications. The system of care of ACS is evolving and constrained by low patients' eligibility and lack of health care infrastructure for reperfusion with attendant high mortality. We recommend urgent robust preventive strategies and local context‐based ACS management guidelines including improved capacity for myocardial reperfusion.

## Sources of Funding

This work was supported by an AstraZeneca Research Grant Trust received by Dr Isezuo.

## Disclosures

None.

## Supporting information

Table S1
Figure S1
