# Supplementary material for: Registry for Acute Coronary Events in Nigeria (RACE‐Nigeria): Clinical Characterization, Management, and Outcome
Source: J Am Heart Assoc. 2021 Dec 22;11(1):e020244. doi: 10.1161/JAHA.120.020244 (PMC9075212; doi:10.1161/JAHA.120.020244)
Supplement: Supplementary file 1 — Table S1 Figure S1 [file JAH3-11-e020244-s001.pdf]

Supplemental Material

Table S1. Factors that increased the Odds of all-Cause Mortality on Univariate Analysis

Variable	OR	95% CI	+P-Value
Socioeconomic status	2.257	1.548-3.376	<0.001
Systolic blood pressure	1.022	0.972-0.987	<0.001
Diastolic blood pressure	1.019	0.970-0.992	0.001
Heart rate	1.028	1.019-1.037	<0.001
STEMI	1.916	0.362-0.754	0.001
Troponin positive	2.294	0.232-0.821	0.010
Random blood sugar	1.007	0.989-0.998	0.002
Renal impairment	3.003	0.213-0.159	<0.001
Left ventricular ejection fraction	1.060	0.927-0.959	<0.001
Left ventricular systolic dysfunction	1.233	1.096-1.388	0.001
Left ventricular diastolic dysfunction	1.632	1.242-2.144	<0.001
Cardiogenic shock	6.098	0.105-0.256	<0.001
Mural Thrombus	3.012	0.188-0.587	<0.001
Cardiac arrest	27.739	0.022-0.098	<0.001
Pulmonary edema	3.390	0.202-0.430	<0.001
Intervention (Thrombolysis/PCI/CABG)	4.425	0.167-0.423	<0.001

+P value derived from univariate binary logistic regression.

ACS: Acute coronary syndrome, PCI: Percutaneous coronary intervention, CABG: Coronary artery bypass graft, OR: Odd ratio, CI: Confidence interval

Figure S1. Algorithm for Diagnosis of ACS.

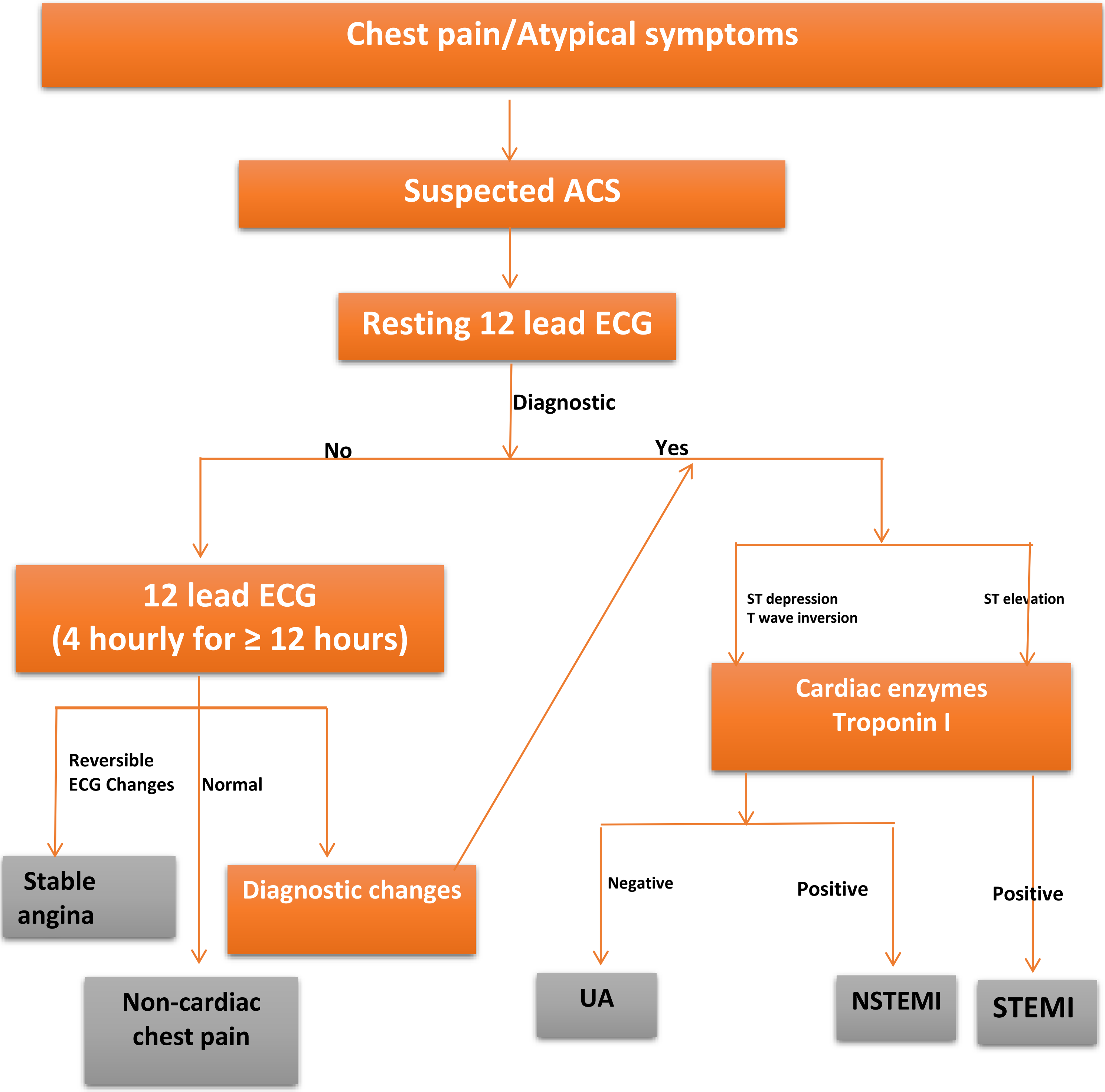

Diagnostic algorithm for Acute coronary syndrome.

ECG: Electrocardiography.
STEMI: ST segment elevation myocardial infarction.
NSTEMI: Non-ST segment elevation myocardial infarction.
UA: Unstable angina.
